# Large-Scale Collection and Analysis of Full-Length cDNAs from *Brachypodium distachyon* and Integration with Pooideae Sequence Resources

**DOI:** 10.1371/journal.pone.0075265

**Published:** 2013-10-09

**Authors:** Keiichi Mochida, Yukiko Uehara-Yamaguchi, Fuminori Takahashi, Takuhiro Yoshida, Tetsuya Sakurai, Kazuo Shinozaki

**Affiliations:** 1 Biomass Research Platform Team, Biomass Engineering Program Cooperation Division, RIKEN Center for Sustainable Resource Science, Tsurumi-ku, Yokohama, Kanagawa, Japan; 2 Integrated Genome Informatics Research Unit, RIKEN Center for Sustainable Resource Science, Tsurumi-ku, Yokohama, Kanagawa, Japan; 3 Kihara Institute for Biological Research, Yokohama City University, Totsuka-ku, Yokohama, Kanagawa, Japan; Harbin Institute of Technology, China

## Abstract

A comprehensive collection of full-length cDNAs is essential for correct structural gene annotation and functional analyses of genes. We constructed a mixed full-length cDNA library from 21 different tissues of *Brachypodium distachyon* Bd21, and obtained 78,163 high quality expressed sequence tags (ESTs) from both ends of ca. 40,000 clones (including 16,079 contigs). We updated gene structure annotations of *Brachypodium* genes based on full-length cDNA sequences in comparison with the latest publicly available annotations. About 10,000 non-redundant gene models were supported by full-length cDNAs; ca. 6,000 showed some transcription unit modifications. We also found ca. 580 novel gene models, including 362 newly identified in Bd21. Using the updated transcription start sites, we searched a total of 580 plant *cis*-motifs in the −3 kb promoter regions and determined a genome-wide *Brachypodium* promoter architecture. Furthermore, we integrated the *Brachypodium* full-length cDNAs and updated gene structures with available sequence resources in wheat and barley in a web-accessible database, the RIKEN *Brachypodium* FL cDNA database. The database represents a “one-stop” information resource for all genomic information in the Pooideae, facilitating functional analysis of genes in this model grass plant and seamless knowledge transfer to the Triticeae crops.

## Introduction

Grasses are major carbon sources for human populations and include commodity plants used for foods and feeds, as well as for biomass. Genome-guided breeding to improve traits is emerging as an approach to achieve sustainable agriculture and to develop renewable energy and materials. Model plant systems are essential to accelerate the necessary gene discovery and for molecular characterization of gene function and the regulatory networks underlying agriculturally important traits, and to promote translational research of crop improvements [1].

*Brachypodium distachyon* (L.) is a model plant for analyzing genetic functions and biological systems in temperate grasses, cool season cereals, and dedicated biofuel crops [2]. The species is characterized by a short life cycle, small plant size, facile transformation, simple growth requirements, and small genome size. *Brachypodium* belongs to the Pooideae subfamily and serves as a model system for major crops, such as wheat, barley, rye, and oat [3]. In 2010, the whole genome sequence of the inbred line Bd21 was released as the first of that of a member of the Pooideae subfamily [4]. *Brachypodium* has subsequently garnered attention and a number of genomic resource projects have been initiated at various institutions [5]. Immediate development of genomic resources is required to utilize this model grass to achieve a number of emerging aspects.

Recent remarkable advancements in genome sequencing approaches in wheat and barley have revitalized genomic platforms for gene discovery in these Pooideae staple crops. The barley (*Hordeum vulugare*) gene-space in a structured whole-genome context was presented by The International Barley Genome Sequencing Consortium as an integrated physical, genetic, and functional sequence assembly [6]. The large, 17-Gb hexaploid genome of bread wheat (*Triticum aestivum*) has been analyzed by 454 pyrosequencing, yielding 94,000–95,000 genes [7]. A comparison of wheat genomic reads with *Brachypodium* genome sequences by these authors demonstrated high-resolution syntenic gene organization. More recently, draft genome sequencing of the A-genome and D-genome donors, *Triticum urartu* and *Aegilops tauschii,* which were performed using high-throughput sequencing, were also published [8], [9]. The recent development of genomic resources in Pooideae crops and analytical tools available in *Brachypodium* facilitates functional analysis of key genes and specific properties of Pooideae grass species. Based on comparative genomics, integration of available genomic datasets in these Pooideae species should synergistically enable us to use genomic features like conserved syntenies to accelerate the gene discovery process as well as complement and enrich our knowledge of structures and functions of genes in this subfamily.

Full-length cDNA libraries and large-scale sequence data sets provide invaluable genomic resources for life science projects in various species [10]–[14]. The sequence resources derived from full-length cDNAs can also help in identifying transcribed regions and structural features, such as transcription units, transcription start sites (TSSs), and transcriptional variants in completed or draft genome sequences [15]–[17].

Full-length cDNAs are also useful for protein expression from entire coding sequences following determination of the three-dimensional (3D) structures of proteins by X-ray crystallography and nuclear magnetic resonance (NMR) spectroscopy [18], and for functional biochemical analyses of expressed proteins in molecular interactions with ligands, proteins, and DNA. Full-length cDNA libraries have also contributed to functional analyses by enabling the creation of over-expression strains used in reverse genetics. For example, the advent of function-based gene discovery via full-length cDNA overexpressor (FOX) gene hunting, which uses full-length cDNA transgenic plants as overexpressors, has enabled high-throughput discovery of functional genes associated with phenotypic changes [19]–[21]. Moreover, large-scale analyses of full-length cDNA sequences and clone resources have been conducted in wheat and barley [22]–[24]. To integrate full-length coding sequence information in wheat and barley, we previously established a database, TriFLDB (http://trifldb.psc.riken.jp), providing functional annotation and associated information based on comparative analysis with other plant species [25]. Along with such advances in collation of sequence resources in Pooidea plants, comprehensive collection of full-length cDNAs from *Brachypodium* could provide a key resource to promote functional analysis and accelerate molecular characterization of Pooideae species.

We have constructed a comprehensive full-length cDNA resource for the *B. distachyon* Bd21 accession. Herein, we report the collection and sequencing of ca. 80,000 full-length cDNAs with their functional annotations. We also performed a comparative analysis among Pooideae transcripts with genomic sequence resources of *Brachypodium* and barley to build an integrated genomic knowledge base. This comparative mapping approach demonstrated that combinatorial use of Pooideae cDNAs could synergistically facilitate the discovery of transcription units and gene structural annotation in Pooideae species. Finally, we established a web accessible database to provide this full-length cDNA dataset and integrated genomic information based on a comparative analysis with wheat and barley.

## Materials and Methods

### Plant Materials

Total RNA was extracted using Plant RNA Reagent (Life technologies, Carlsbad, CA, USA) from the inbred line *B. distachyon,* Bd21, under the conditions depicted in Table 1. The plants used for RNA extraction from normal tissues of each developmental stage were grown in soil pots in greenhouses or by hydro-culture in a growth chamber. The greenhouse and growth chamber were maintained at 16-h light:8-h dark at 22°C. The callus tissue was induced and cultured on Gellan Gum plates of Murashige & Skoog medium including vitamins (Duchefa Biochemie, B.V., Haarlem, The Netherlands) with 3% sucrose, 2.5 µg/ml 2,4-D. For stress tissues, the tissue samples were also collected from plants grown in hydro-culture in a growth chamber maintained at 16-h light and 8-h dark at 22°C.

**Table 1:** Collection of RNA samples for constructing a *Brachypodium* full-length cDNA library.

	Tissue	Treatment	Growth condition
Normal tissues	Seed at germination		Hydro-culture, growth chamber
	Shoot		Hydro-culture, growth chamber
	Leaf at vegetative stage		Pot soil, green house
	Leaf after flowering		Pot soil, green house
	Root		Hydro culture, growth chamber
	Crown		Hydro culture, growth chamber
	Spikelet at flowering		Pot soil, green house
	Spikelet (DAP1–5)		Pot soil, green house
	Spikelet (DAP7–10)		Pot soil, green house
	Spikelet (DAP20–30)		Pot soil, green house
Callus			Culture on agarose gel, growth chamber
Stress tissues	Leaves at 2 weeks after germination	250 mM NaCl 5 h	Hydro-culture, growth chamber
		250 mM NaCl 24 h	
		100 µM ABA 5 h	
		100 µM ABA 24 h	
		Cold stress 4°C 5 h	
		Cold stress 4°C 24 h	
		Drought stress (on filter paper) 5 h	
		Drought stress (on filter paper) 24 h	
		Heat shock 42°C 2 h	
		Wounding 1 h	

### RNA Preparation and Construction of Full-length cDNA Library

A full-length cDNA library was constructed from the poly(A)^+^ RNA by the biotinylated CAP trapper method, using trehalose-thermoactivated reverse transcriptase [26]. The resulting double-stranded cDNAs were digested with *Bam*HI and *Xho*I, and ligated into the *Bam*HI and *Sal*I sites of a Lambda FLC-III vector [27].

### Sanger Sequencing and EST Analysis

Transformed bacteria were randomly selected. Plasmid DNA from each FL cDNA clone was directly amplified from 384 bacterial cultures in a glycerol stock plate by the rolling circle amplification (RCA) method using an Illustra TempliPhi DNA Amplification kit (GE Healthcare, United Kingdom). End-sequencing of 39,936 clones was performed by the Sanger method using ABI 3730 capillary sequencers (Applied Biosystems, Foster City, CA, U.S.). The T7 promoter primer (5′-TAATACGACTCACTATAGGG-3′) and the T3 promoter primer (5′-ATTAACCCTCACTAAAGGGAA-3′) were used for forward and reverse sequencing, respectively. Raw sequence data were base-called with the KB basecaller software of the ABI 3730 sequencer. Low-quality regions were trimmed based on Phred quality score (QV≤15), and data with more than 300 bp untrimmed regions were retained for additional clean-up. SeqClean (http://compbio.dfci.harvard.edu/tgi/software/) was used to trim the vector sequence and polyA tail with default parameter settings. The paired forward and reverse ESTs for each clone were assembled by CAP3 [28] with the default parameter settings; the CAP3 contigs and singlets were classified into completely sequenced transcripts and partial cDNA sequences, respectively.

### Mapping and Structural Annotation

We mapped the *B. distachyon* ESTs and those assembled contigs to update genome annotation in structures of transcription units using the Program to Assemble Spliced Alignments (PASA) pipeline [29] with the unmasked Bd21 genome sequence dataset. The structural annotation data were downloaded from Phytozome (ver.8.0) and MIPS (ver.1.2), both of these data sets include 31,029 gene models of identical identifiers with some differences in those transcriptional structures. The cDNA sequences were mapped to the genomic sequence using the GMAP mapping tools in the PASA pipeline with the default parameter settings [30]. Then, we updated the structural annotations of *Brachypodium* genes together with gene annotations from Phytozome 8.0 and MIPS 1.2, respectively using the annotation update method in the PASA pipeline.

### Functional Annotations

To annotate the RBFL cDNA with predicted gene functions, we searched the sequence data against the following protein and nucleotide datasets using the BLAST algorithm [31]: the nr protein database of NCBI (ftp://ftp.ncbi.nih.gov/blast/db); UniProt/trembl of EBI (http://www.uniprot.org/downloads); the protein and cDNA data of rice derived from RAP-DB v. 2 (http://rapdb.dna.affrc.go.jp/) and the MSU Rice Genome Annotation Project (http://rice.plantbiology.msu.edu/); as well as the protein and cDNA data present in TAIR release 10 (ftp://ftp.arabidopsis.org/home/tair/Sequences/blast_datasets/).

To find possible functional descriptions, all similarity searches with BLASTX and BLASTP against protein datasets were performed with a threshold e-value of less than 1e-5, and the top scoring hit for each query was selected. The threshold of an e-value of less than 1e-5 has been widely used in blast-based functional prediction of cDNAs in plants [32]–[34].

The definition strings used for the similarity searches of each database were assembled as a keyword database to allow users to specify queries with keywords to retrieve relevant gene information from the RIKEN *Brachypodium* full-length cDNA database (RBFLDB). Conserved domains in the deduced protein sequence of each *Brachypodium* gene were identified with InterProScan and the InterPro database (http://www.ebi.ac.uk/interpro/). The blast-based similarity search result against *Arabidopsis* genes and domain search results were used to assign GO terms to each of the *Brachypodium* genes, which are also available as search terms for the RBFLDB entries. The GOSlimeViewer of AgBase web site (http://www.agbase.msstate.edu/cgi-bin/tools/goslimviewer_select.pl) was used to summarize GO functional annotations. Links to each of the original datasets interrelated with RBFLDB entries are provided on the RBFLDB web-interface.

### *cis*-Motif Search

To discover *cis*-regulatory motifs located in the promoter regions of each gene model, *cis*-motif sequences from the PLACE database (version 30, 469 entries) (http://www.dna.affrc.go.jp/PLACE/), AGRIS database (http://arabidopsis.med.ohio-state.edu/AtcisDB/bindingsites.html), and the previously reported stress responsive *cis*-motifs [35] were used. The retrieved sequence patterns of each cis-motif were searched against the −500, −1000 and −3000 bp ranges upstream from the putative transcription start site in the updated gene annotation of the *Brachypodium* genome sequence using a custom Perl script.

To investigate over represented cis-motifs in functionally classified gene groups, we used MapMan ontology annotation of *Brachypodium* genes (http://mapman.gabipd.org/web/guest/download). To evaluate enriched cis-motifs in each of the gene groups in the MapMan ontology, computation of the overrepresentation test and its significance were performed by a Z-test [36]. The Z-test was conducted for gene groups including more than 200 gene models. The associated p-value of each cis-motif in each of the gene groups was commutated based on 100 random samplings with 100 replicates and was then corrected using the Bonferroni correction with 10 replications.

### Sequence Resources in Triticeae Species

We retrieved genomic sequence datasets of wheat and barley from the public domains to integrate Pooideae genomic information. A dataset of full-length barley cDNAs was retrieved from Genbank (accession No. AK353559– AK377172) and TriFLDB (http://trifldb.psc.riken.jp/). Genomic sequence assemblies were downloaded from the MIPS barley genome database (ftp://ftpmips.helmholtz-muenchen.de/plants/barley/public_data/). A dataset of full-length cDNAs of wheat was retrieved from TriFLDB. The shotgun sequence assembly obtained by Roche 454 sequencing of wheat (UK 454 survey) was downloaded from the MIPS wheat genome database (ftp://ftpmips.helmholtz-muenchen.de/plants/wheat/UK_454/). The updated gene models using RBFL cDNAs with MIPS 1.2 annotation were used in the comparative mapping analysis as a query of *Brachypodium*. Cross-species mapping using cDNA and gene model datasets for querying the *Brachypodium* and barley genome was performed by sim4 with default parameter settings [37], followed by a BLASTN search, with e-value cut-offs of less than 1e-20.

### Database Construction

All of the generated datasets were stored in a MySQL database. The user interfaces of the web-accessible database, RBFLDB were developed using Perl CGI in combination with Java script. The description strings of each associated annotation, based on homologous genes, protein domains, cis-motifs found in promoter regions, and assigned Gene Ontology terms, were used to search targets in the keyword search. The NCBI www BLAST server was implemented in the RBFLDB to provide a sequence similarity search interface against the sequence dataset of RBFL cDNAs as well as datasets of related organisms. The Generic Genome Browser (Gbrowse) was implemented in the database with *Brachypodium* genome annotations released by Phytozome 8 and MIPS 1.2, as well as those in barley released by MIPS and Ensembl Plant. Information regarding all of the sequence datasets used to generate datasets in the RBFLDB is summarized in Table S1.

## Results and Discussion

### Cloning and Sequencing

We used the biotinylated CAP trapper method [26] to construct a full-length cDNA library of *Brachypodium distachyon* Bd21 from various normal tissues of developmental organs as well as root and leaf tissues, under various abiotic stresses (Table 1). The λFLCIII vector [27], which accommodates cDNAs in a broad range of sizes and is useful for the high-efficiency cloning of long cDNA fragments, was used to construct the cDNA library. The approximately 40,000 recombinant clones were randomly selected and sequenced from both ends using the Sanger method. We obtained 39,358 and 38,805 sequences from the forward and reverse directions, respectively, and from among the 40,000 clones we obtained the forward and reverse sequences of a total of 38,446 clones (Table 2). We also assembled the forward and reverse reads to produce 16,079 contigs. A total of 94,242 sequences have been deposited in the DDBJ public sequence database (accession numbers: HX789325–HX867487 for reads deposited in the EST division and AK424275–AK440353 for contigs deposited in the HTC division). We have named these “RBFL” (RIKEN *Brachypodium* Full-Length) cDNAs. Mapping analysis of the full-length cDNAs to the Bd21 genome showed that more than 96% (91,309) of reads were mappable. To our knowledge, this study is the first large scale collection of full-length-enriched cDNAs in *Brachypodium*, and the sequence datasets and clones should play significant roles in functional analysis of *Brachypodium* genes as well as for comparative gene discovery in grass plants.

**Table 2:** Sequence resources to update structural gene annotation in the *Brachypodium* genome.

		No. sequences	Min. length (bp)	Max. length (bp)	Mean length (bp)
Clones sequenced from both ends		38,446			
Full-length cDNA reached from both ends (Contigs)		16,079	150	1152	808.9
Partial full-length cDNAs sequences (ESTs)	Sanger FL cDNA ESTs 5′	39,358	103	640	581.3
	Sanger FL cDNA ESTs 3′	38,805	105	613	556.3
Total sequences in the structural annotation		94,242			
Total sequences mapped onto the Bd21 genome		91,309			

### Improvement of *Brachypodium* Structural gene Annotation

Sequence datasets of full-length cDNAs facilitated accurate structural gene annotation and identification of novel transcription units in a number of model organisms. To our knowledge, there are two publicly available gene annotations for *Brachypodium distachyon* Bd21; one was released from Phytozome 8.0 and another from MIPS 1.2, and each contains the same number of annotated gene models on identical loci, with slightly different annotations in the UTRs. We applied the RBFL dataset to update these structural annotations of the *Brachypodium* genome. By mapping in the PASA pipeline, 10,513 and 10,500 non-redundant gene models in updated annotations of Phytozome 8.0 and MIPS 1.2 corresponded to one or more ESTs and/or contigs of full-length cDNAs (Table 3). This result indicated that one-third of transcripts of known gene models could be captured as full-length cDNA clone resources. Structural annotation analysis using a eukaryotic genome annotation tool (PASA) with the *Brachypodium* full-length cDNA sequences (ESTs and contigs) showed 31,009 gene models taken from both of the existing annotations, and 681 and 679 newly designed gene models (including known loci) against Phytozome 8.0 and MIPS 1.2, respectively (Table 4). Furthermore, the structural full-length cDNA-based annotation suggests that 20 neighboring gene model annotations should be fused into single transcripts. In total, the full-length cDNA-based structural annotation updated the previous annotations of 31,690 and 31,688 gene models from Phytozome 8.0 and MIPS 1.2, respectively (Table 4).

**Table 3:** *Brachypodium* gene models corresponding to RBFL sequences.

	Phytozome 8.0	MIPS1.2
Gene models with RBFL ESTs	6,432	6,415
Gene models with RBFL full-read contigs	4,081	4,085
Total	10,513	10,500

**Table 4:** Statistics of updated structural gene annotation in the *Brachypodium* Bd21 genome.

	Original annotation	
	Phytozome 8.0	MIPS1.2
*Brachypodium* gene annotation	31,029	31,029
Updated annotation based on the PASA analysis	31,690	31,688
Fused to one gene model (removed from previous annotation)	20	20
Remaining gene models	31,009	31,009
Remaining gene models with modification	6,241	6,180
Remaining gene models with modification in CDS regions	719	717
Remaining gene models with modifications in 5′UTRs	5,399	5,340
5′UTR addition	3,222	3,195
5′UTR extension	2,038	2,021
Other modifications in 5′UTRs	139	124
Remaining gene models with modifications in 3′UTRs	2,606	2,571
3′UTR addition	965	967
3′UTR extension	1,471	1,451
Other modifications in 3′UTRs	170	153
Newly defined gene models	681	679
Novel gene models (novel loci and TUs)	362	362
Newly added isoforms	304	302
Fused	10	10
Others	5	5

Other modifications in UTRs include UTR removal, abbreviation, and any other structural changes.

**Figure 1:**
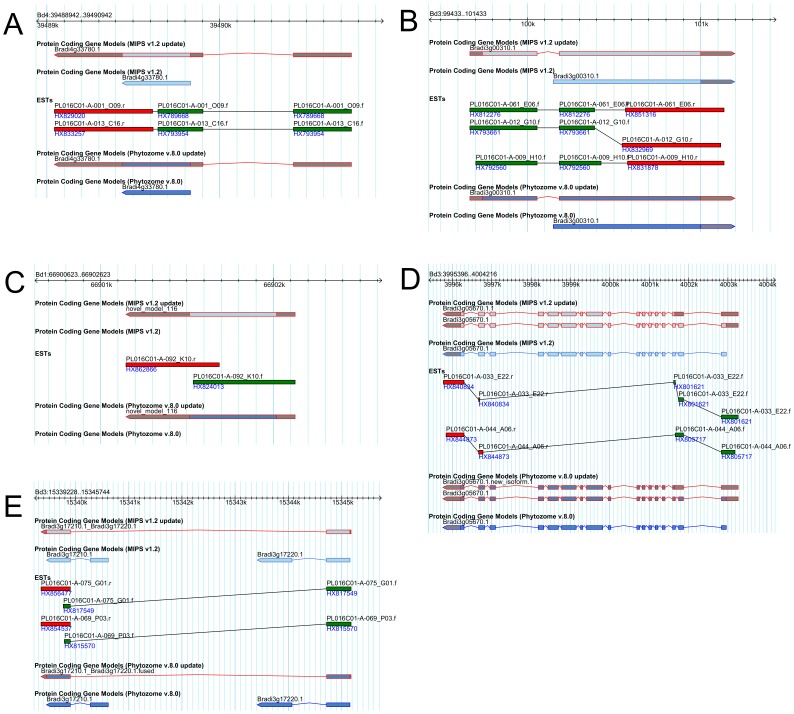
Examples of updated structural annotation based on full-length cDNA sequences in the *Brachypodium* genome; UTR addition (A), newly identified exon (B), newly identified transcription unit (C), newly identified gene model (D) and fused gene model (E). Forward and reverse sequence reads of the *Brachypodium* full-length cDNA are represented in green and red color, respectively. Modified gene structures are indicated with red lines.

In the ca. 10,000 gene models supported by the full-length cDNAs, more than 60% (6,241 in Phytozome 8.0 and 6,180 in MIPS 1.2) showed at least one structural modification (Table 4). The combinations of structural modifications in both UTRs and CDS are summarized in Table S2. About 86% (5,399 in Phytozome 8.0 and 5,340 in MIPS 1.2) of modified gene models carried modifications in their 5′UTRs, such as additions and extensions. Thus, most sequences were derived from intact mRNA with transcription start sites and represent the remarkable contribution of full-length cDNAs to annotate translation start sites and 5′UTRs (Figure. 1A, B). Accurate annotation of 5′UTRs is essential to identify entire transcription units and to analyze regulatory elements in the upstream regions of coding sequences, such as promoters, upstream ORFs, and alternative translation start sites and/or alternative first exons. About 42% of modified gene models (2,606 in Phytozome 8.0 and 2,571 in MIPS 1.2) showed modifications such as addition and extensions of 3′UTRs (Figure 1A). Comparative distributions of UTR length of the *Brachypodium* gene models within original annotations of MIPS 1.2 and Phytozome 8 and updated annotations of those with RBFL sequences showed measurable additions and extensions in length of both UTRs (Figure S1). Recent massive sequencing approaches to polyadenylation sites revealed extensive alternative polyadenylation in the 3′UTRs of genes in various organisms [38]. Alternative cleavage and polyadenylation are emerging as an important layer of gene regulation, as they generate transcript isoforms that differ in their 3′UTRs, thereby modulating gene responses to 3′UTR-mediated regulation. Therefore, accurate annotation of 3′UTRs is also essential reference information for analyzing transcriptional properties in the UTRome.

The structural annotation identified new transcription units, including novel gene loci, new isoforms in known loci, and fused transcription units (Table 4). The numbers of newly defined transcription units were 681 and 679 in Phytozome 8.0 and MIPS 1.2, respectively. Among them, 362 gene models were newly identified that were allocated to genomic regions without gene loci in previous *Brachypodium* annotations of MIPS1.2 and Phytozome 8. Comparative mapping analysis using wheat and barley full-length cDNAs also showed putative orthologous transcripts of these novel loci (Figure 1C), suggesting that they are conserved and transcribed in Pooideae plants (Figure S4). The rest of the newly defined transcription units with gene loci annotated in MIPS1.2 and Phytozome 8, 304 and 302 respectively, were added to previously annotated loci as new splicing isoforms, with some modifications in coding sequence structure (Figure 1D). From paired long ESTs sequenced from both cDNA ends, we were also able to identify 10 gene models, corresponding to 20 neighboring gene models from previous annotations. For example, in Figure 1E, the RBFL clones, PL016C01-A-075_G01 and PL016C01-A-069_P03 include an entire open reading frame that is identical to the Refseq entry XP_003571495 putatively encoding PP2-B10-like F-box protein. On the other hand, with previous annotations, the 5′ half of Bradi3g1720.1 and the 3′ half of Bradi3g1710.1 are respectively matched to the N- terminal and C-terminal half of the refseq entry; each of these encode a truncated Phloem protein 2 (PP2) domain. These facts suggest that Bradi3g1720.1 and Bradi3g1710.1 should be fused into a single transcription unit and its splicing form altered.

To date, many projects involving full-length cDNA sequencing have been conducted in eukaryotic species. After completion of genome-sequencing and full-length cDNA projects in target species such as human, mouse, and rice, “jamboree-style” annotation meetings have been orchestrated such as H-inv (Human Invitational Annotation project) [11], [14], FANTOM (Functional Annotation Of Mouse) [12], and RAP (Rice Annotation Projects) [39], [40]. Accurate gene structural annotations in these species have played essential roles in various studies in post genome projects, and associated databases have become hubs of information resources for each species. Recently, accurate gene structural annotations have been also essential as reference genome annotation for various next-generation sequencing applications with short-read mapping, such as RNA-seq, exome sequencing and Chip-seq.

Our sequence data resource of *Brachypodium* transcription units should contribute to the accurate prediction of genetic structures in the *Brachypodium* genome. The use of *Brachypodium* full-length cDNA sequences will accelerate the process of manual refinement of computational annotations for this model grass.

### Functional Annotations of *Brachypodium* Genes in Association with Full-length cDNAs

All annotated *Brachypodium* gene models, including the newly identified gene loci, were searched against various sequence datasets to obtain clues as to gene function (Figure S2). Sequence datasets, such as the annotated protein datasets of Arabidopsis and rice (RAP-DB and TIGR), as well as representative nonredundant protein data repositories (nr of NCBI and UniProt of EBI) were used to collect putative functional descriptions for each gene model. For domain-based functional annotation, the deduced protein data were subjected to a domain search, followed by classification of gene models into protein families and assignment of Gene Ontology terms to *Brachypodium* gene models and corresponding RBFL cDNAs (Figure S3).

*Cis*-regulatory elements, which are the binding sites for transcription factors located in promoter regions, are the functional elements that determine the timing and location of transcriptional activity. Extensive promoter analyses have identified a large number of *cis*-elements, which are important molecular switches in the transcriptional regulation of a dynamic network of gene activities controlling various biological processes, such as abiotic stress responses, hormone responses, and developmental processes. The updated structural annotations, particularly in the 5′UTRs, allowed us to explore promoter sequences to annotate *cis*-regulatory elements on the genome sequence. The PLACE and AGRIS databases have consolidated all published *cis*-motifs. In addition, known stress-responsive *cis*-motifs have also been reported.

**Figure 2:**
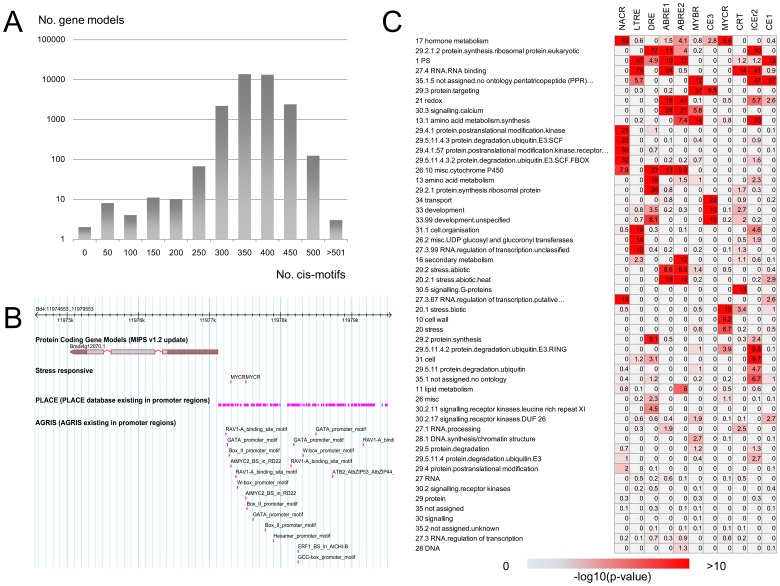
Promoter architecture in the *Brachypodium* genome analyzed using updated gene structural annotation. Distribution of all *cis*-motifs searched in the −3000 bp promoter regions from putative translation start sites (A). An example of implemented *cis*-motif data found in the −3000 bp promoter regions, which is scrutinized on a genome browser in the RBFLDB (B). Enriched stress responsive *cis*-motifs in the −1000 bp promoter regions of genes classified into functional categories based on MapMan ontology, which are hierarchically clustered into functional categories and by the *cis* motifs (C).

To facilitate the functional characterization of *Brachypodium* genes, we retrieved the promoter regions for all annotated gene models based on our updated annotation. The −3000 bp promoter regions from the updated transcription start sites were subjected to extensive *in silico* analyses to search for all known *cis*-regulatory motifs. We searched, in total, 580 (12 published stress responsive motifs, 99 motifs from the AGRIS database and 469 motifs from the PLACE database) known cis-motifs in the promoter regions and annotated those genomic positions. Almost all promoter regions (99.67%) of gene models showed more than 300 sequence patterns matching to the known cis-motifs (Figure 2A). All datasets of the *cis*-motif search were added into the web-accessible database and genome browser to integrate promoter information with the RBFL cDNAs and to visualize the related genomic architecture together with updated structural gene annotation (Figure 2B).

Integration of functional gene annotation and *cis*-motifs profile allowed us to analyze relationships between the contexts of gene functions and properties of cis-motifs that may be involved in particular transcription networks. We performed enrichment analysis of *cis*-motifs in classified gene sets to define ontologies of gene functions in 11 stress-responsive *cis*-motifs. The hierarchically clustered heat map of enriched *cis*-motifs and the ontologies of gene functions represented co-enriched stress responsive *cis*-motifs located in the promoters of genes classified in particular ontologies. For example, LTRE, DRE, ABRE1, ABRE2 and CE1 motifs are co-enriched in the gene group of photosynthesis. Additionally, the ABRE1 and ABRE2 motif is significantly shared in the promoters of genes involved in abiotic stress related functions, especially those involving heat stress (Figure 2C).

A combination of integrated analysis based on gene functional annotations and *cis*-motif analysis could facilitate the systematic functional predictions of *Brachypodium* genes. These combinatorial datasets, together with full-length cDNA clones, should provide an effective context filter to dissect elaborate transcription networks and to narrow down genes involved in particular biological functions, which can strongly assist gene discovery in the model grass.

Sequence-specific DNA-binding TFs are key molecular switches that control or influence development, growth, cell division, and responses to environmental stimuli in a cell or whole organism. We have previously released the GramineaeTFDB database (http://gramineaetfdb.psc.riken.jp) [41]) to integrate all putative TFs from six grass species: *Brachypodium distachyon*, maize, rice, sorghum, barley, and wheat, which includes relevant information for comparative genomics and functional genomics in regard to transcription factors. The RBFL sequences were assigned to *Brachypodium* transcription factors annotated in the GramineaeTFDB, and 657 (31.4% of all putative *Brachypodium* gene models encoding TFs) non-redundant gene models were classified into the plant TF repertoire (Table 5). Detailed functional annotation of TF-encoding genes provided by the GramineaeTFDB and the corresponding full-length cDNA clones should facilitate the molecular characterization of transcriptional networks. Furthermore, extraction of the upstream regions of the updated translation start sites, combined with direct evidence from, for example, ChIP-Seq or genome-wide one-hybrid studies, will accelerate the discovery of regulatory networks in the transcriptome of this model grass.

**Table 5:** Gene models encoding transcription factors cloned as full-length cDNA.

TF	GramineaeTFDB	RBFL	TF	GramineaeTFDB	RBFL
(R1)R2R3_Myb	104	27	HSF	30	14
ABI3VP1	48	4	JUMONJI	19	2
AP2_EREBP	153	62	LFY	1	0
ARF	36	11	LIM	20	8
ARID	10	4	LUG	5	1
Alfin-like	12	8	MADS	75	8
Aux_IAA	38	22	MBF1	3	2
BBR-BPC	4	1	Myb_related	45	19
BES1	7	3	NAC	103	30
C2C2_Zn-CO-like	34	20	Nin-like	15	1
C2C2_Zn-Dof	27	9	PHD	178	43
C2C2_Zn-GATA	24	10	PLATZ	14	4
C2C2_Zn-YABBY	13	4	PcG	51	5
C2H2_Zn	102	24	S1Fa-like	2	2
C3H-TypeI	78	30	SBP	18	4
CAMTA	10	2	SRS	4	1
CCAAT_Dr1	1	1	TCP	21	5
CCAAT_HAP2	12	5	TUB	12	7
CCAAT_HAP3	16	4	Trihelix	8	4
CCAAT_HAP5	13	4	ULT	1	0
CPP	11	5	VOZ	2	1
E2F_DP	8	2	WRKY_Zn	89	23
EIL	6	3	Whirly	2	1
GARP_ARRB	9	5	ZIM	19	14
GARP_G2-like	56	18	atypical_MYB	36	14
GRAS	47	10	bHLH	151	41
GRF	28	1	bZIP	96	44
GeBP	16	5	zf-HD	16	2
HB	101	41	zf-TAZ	9	4
HMG-box	11	6	(ambiguous)	(11)	(2)
HRT	1	0			
			Total	2,092	657

### Data Integration with Triticeae Sequence Resources

**Figure 3:**
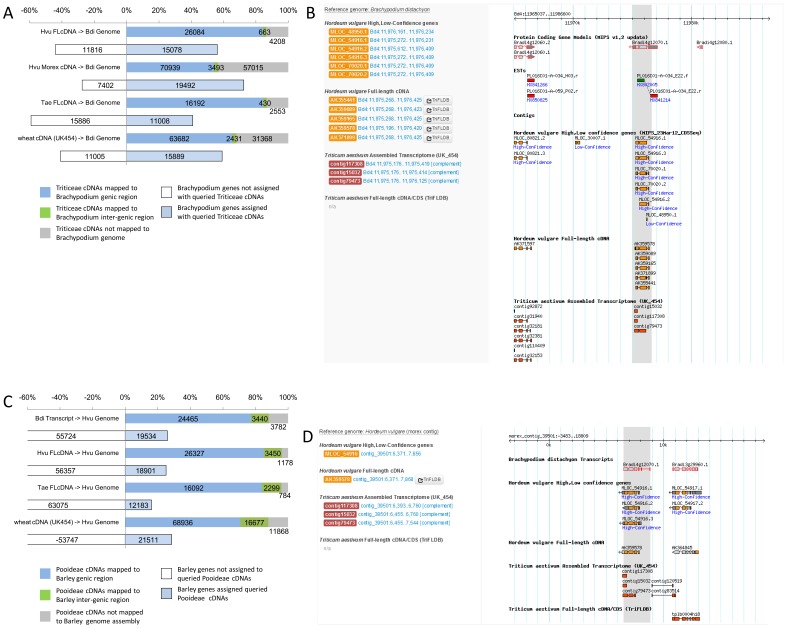
Comparative mapping of Pooideae cDNAs or gene models to the *Brachypodium* genome or barley genome assembly. The updated gene models of *Brachypodium* from MIPS 1.2 annotation using RBFL cDNAs were used for this comparative mapping analysis. Summarized results of comparative cDNA mapping of barley and wheat cDNAs to *Brachypodium* genome. Numbers of queries of barley full-length cDNAs (Hvu FLcDNA), gene models annotated in the barley Morex genome (Hvu Morex cDNA), wheat full-length cDNAs (Tae FLcDNA) and wheat gene models from a shotgun genome assembly (wheat cDNA(UK454)), respectively, mapped to the genic, or inter-genic regions of the *Brachypodium* genome (Bdi Genome), or not mapped, are represented. Numbers of *Brachypodium* genes located on the region with mapped Triticeae cDNAs or gene models are also shown (A). An example of mapping results of Triticeae cDNAs with *Brachypodium* gene annotation (B). Summarized results of comparative cDNA mapping of barley, numbers of queries of *Brachypodium* transcripts (Bdi Transcript), barley full-length cDNAs (Hvu FLcDNA), wheat full-length cDNAs (Tae FLcDNA), and wheat gene models of a shotgun genome assembly (wheat cDNA (UK454)), respectively, mapped to the genic, or inter-genic regions of the barley Morex genome assembly (Hvu Genome), or not mapped, are represented. Numbers of barley genes located on the region with mapped the queried Pooideae cDNAs or gene models are also shown (C). An example of mapping results of the Pooideae cDNAs with barley gene annotation (D).

Recent extensive progress of sequencing projects in the Triticeae plants, wheat and barley, has allowed us to access comprehensive sequence resources of full-length cDNAs and genomic sequence assemblies. Combinatorial use of available sequence resources in Pooideae should accelerate gene discovery and knowledge transfer between *Brachypodium* and these Triticeae crops. To achieve significant data integration of sequence resources in Pooideae, we performed a comparative analysis of *Brachypodium*, wheat, and barley, based on full-length cDNAs and genomic assemblies. To establish the interrelationships of Pooideae transcripts and genomic sequences, comparative mapping analysis was performed using available comprehensive cDNA sequences from each species of wheat, barley, and *Brachypodium* as queries against the genomic sequences of *Brachypodium* and barley. In this analysis, with *Brachypodium*, we used the updated annotation (Bdi Genome) and cDNA sequences (Bdi Transcript) by using the RBFL dataset with MIPS1.2 annotation as the query and the reference genome. With barley, we used the publicly available full-length cDNA datasets of cultivar Harna Nijo (Hvu FLcDNA) and predicted transcripts in the genomic assembly of cultivar Morex (Hvu Morex cDNA) as the query. We used the sequence dataset of the genomic assembly of cultivar Morex as the reference genome (Hvu Genome). With wheat, we used wheat full-length cDNA dataset from TriFLDB (Tri FLcDNA) and an assembled cDNA dataset associated with the wheat genome shotgun data (wheat cDNA (UK454)). The results of this comparative mapping analysis are summarized in Figures 3A and 3C.

Of the queried wheat entries, about 65.3% (63,682) of cDNA assemblies and 84.4% (16,192) of full-length cDNAs were mapped to the genic regions of *Brachypodium* genome, and those were assigned to 59.1% (15,889) and 40.9% (11,008), respectively, of non-redundant genes in the updated annotation of the *Brachypodium* genome (Figure 3A). With barley transcripts, 84.3% (26,084) of full-length cDNA of the Haruna-nijo cultivar [23], [24] and 54.0% (70,939) of cDNAs of predicted genes annotated in the Morex cultivar genome assembly were mapped to the *Brachypodium* genome; these genes corresponded to 65.0% (15,078) and 74.5% (19,492), respectively, of the annotated *Brachypodium* genes (Figure 3A). In total, the wheat and barley cDNA sequences were assigned to 21,055 non-redundant genes (78.3%) among the *Brachypodium* genes, including 132 genes newly identified in this study (Table S3, Figure S4). Although most entries were allocated to the annotated gene regions of the *Brachypodium* genome, some were allocated to the non-annotated regions. These results suggest that there are potential transcription units of *Brachypodium* with conserved homology to cDNAs in wheat and barley, which have not yet been annotated. (Figure S5). Integrated use of cDNA information in gene prediction in Pooideae species could facilitate discovery of novel transcription units in *Brachypodium*.

The cDNA datasets of *Brachypodium*, barley and wheat were also mapped to the barley Morex genomic contigs, which includes 24,243 high confidence and 51,015 low confidence gene loci. The gene loci in the Morex genome were predicted based on publicly available barley full-length cDNAs and RNA-seq datasets, and were then filtered for high- and low-confidence predictions based on certain criteria for sequence homology to other angiosperm proteins [6]. About 77.2% (24,465) of the *Brachypodium* transcripts, 85.1% (26,327) of the barley full-length cDNAs, 83.9% (16,092) of the wheat full-length cDNAs, and 70.7% (68,936) of the wheat cDNA assembly were mapped to the genic region of barley genomic contigs. Those mapped Pooideae cDNAs were assigned to about 26% (19,534), 25.1% (18,901), 16.2% (12,183) and 28.6% (21,511), respectively, of the predicted genic regions of annotated genes in the barley genomic assembly (Figure 3C). Some of these Pooideae cDNAs were also mapped to inter-genic regions; for example, about 10% of queried cDNAs of *Brachypoidum* and barley were mapped here. In this comparative mapping analysis with the barley genome, in total, at least one Pooideae cDNA sequences were assigned to 32,407 non-redundant genes (43.1%) among predicted genes in the barley genomic assembly (Table S4). This suggests that about 32,000 barley genes could corresponded to some comparable Pooideae cDNAs encode putative orthologs among Pooideae species. These comparative mapping data suggest that integrated use of cDNA information in gene prediction in Pooideae species should synergistically facilitate discovery of novel transcription units in *Brachypodium* as well as barley.

The comparative mapping datasets allowed us to immediately identify full-length cDNAs of putative orthologous genes in counter species of Pooideae, and to compare gene structures conserved among putative orthologs. The integration of comprehensive full-length cDNA resources should be useful for annotating the genomes of the Pooideae and for comparative studies and yielding putative orthologous genomic assemblies that are comparable among Pooideae species. This data integration allows a user to search and browse putative orthologous full-length cDNAs and genomic assemblies of barley, as well as a wealth of information on *Brachypodium* (Figure 3B, D).

Accumulation of the genomic sequences in Pooideae plants is currently progressing [7]–[9]. Therefore, it is important to establish an integrated information resource to provide access to comparable datasets of related organisms. Our comparative analysis is the most comprehensive use of cDNA sequence resources in Pooideae plants to date, and represents orthologous relationships among transcribed sequences among the Pooideae plants. Our results suggested that, although collections of full-length cDNA sequences and clones in Pooideae are still incomplete, a resource integrating this information should not only improve gene annotation, but also gene discovery studies in Pooideae plants. The integrated cDNA information resource given here could provide cross-reference information to improve gene annotation and infer shared orthologs and specific or diverged genes among the Pooideae subfamily.

### Database of Full-length Brachypodium cDNAs

All the annotation data for *Brachypodium* gene models and corresponding RBFL cDNAs generated in this study were integrated into the RBFL cDNA database (RBFLDB, http://brachy.bmep.riken.jp/ver.1/index.pl). The web-based user interface allows one to search and browse information on each of the entries in the RBFLDB (Figure S6).

To access full-length cDNA information, RBFLDB provides a web-based search interface enabling keyword and sequence similarity searches. It is possible to search with keyword strings from BLASTed definitions or with identifiers from protein domains found by InterProScan and the assigned GO terms. In addition, our website provides a *cis*-motif search function, which enables searches for all types of *cis*-motifs provided by the PLACE and AGRIS databases in the promoter region of any annotated gene model and/or the search for gene models that contain the *cis*-motif(s) of interest. The BLAST service allows users to perform a homology search against multiple-sequence datasets. The database for this BLAST service consists of sequence datasets of RBFL cDNA reads and nucleotide and amino acid sequence datasets of genes with updated structural annotation in this study, as well as available full-length cDNA sequence resources for wheat and barley, annotated genes in rice, corn, sorghum, and *Arabidopsis*. These search interfaces provide users with effective access to RBFL cDNA entries and updated gene annotations by using various types of queries that are also used in the databases for other plant species. Users can conveniently access detailed information on full-length cDNA clones, gene annotation (including gene structure, cDNA, and protein sequences of corresponding genes), domain structure predicted by InterProScan, promoter regions, domain alignments, and GO annotation terms derived from InterProScan and comparative analysis with their putative homologs of *Arabidopsis*. A synopsis of the results of the similarity search against various sequence resources is shown on the web interface, which allows researchers to determine the annotation status of the searched entries and the predicted annotation of the most likely counterparts in other databases. This should help users to build hypotheses that are related to gene function. Information on the *cis*-elements located in the promoter region of each gene model is accessible on the detailed page of each gene model. In combination with other functional annotations, these data can facilitate systematic functional predictions of *Brachypodium* genes. The RBFLDB provides more updated structural annotation compared to MIPS 1.2 and Phytozome 8.0. Users can compare transcriptional structures and sequences of cDNAs and proteins in each of the gene models in the detail page, and can also download updated annotation files from the download page.

To visualize predicted exon-intron structures of *Brachypodium* genes, *cis*-motifs on promoter regions and compare genomic features of *Brachypodium* genes with genomic sequences in barley, the Generic Genome Browser (Gbrowse; Donlin, 2007) was used in the RBFL cDNA database. Using the *Brachypodium* Gbrowse interface, users can compare structural annotations among Phytozome 8.0, MIPS 1.2, and our updated annotations with RBFL cDNA reads. Furthermore, the *Brachypodium* Gbrowse consists of annotation tracks for comparative mapping against full-length cDNAs of wheat and barley, which should help to confirm conserved gene structures among orthologs in the Pooideae species.

The RBFLDB should function as a “one-stop” information resource for *Brachypodium* genes and for all genomic publications in Pooideae. The full-length cDNAs, genomic sequences, and functional annotations of the model species increase the ability to cross-reference between Pooideae species, thereby facilitating knowledge exchange to support to comparative grass genomics.

## Supporting Information

Figure S1 **Distribution of UTR length in annotations of MIPS1.2, updated MIPS1.2 with RBFL, Phytozome8 and updated Phytoaome 8 with RBFL.** The length of 3′ UTR (A) and those of 5′UTR (B) are represented.
(TIF)

Figure S2 **Similarity search results of** ***Brachypodium*** **gene models, including newly identified gene models, against various sequence databases.**
(TIF)

Figure S3 **Proportion of** ***Brachypodium*** **genes cloned as full-length cDNAs in a functional classification based on the GO slim category.** The represented data is for category in the generic molecular function.
(TIF)

Figure S4 **An example of newly identified transcription units based on the RBFL cDNAs, which are supported by homologous cDNAs of barley and wheat.**
(TIF)

Figure S5 **An example of potentially transcribed regions of** ***Brachypodium*** **with conserved homology to cDNAs in wheat and barley.**
(TIF)

Figure S6 **Overview of RBFLDB content and user interfaces.** RBFLDB provides various types of search interfaces to access RBFL cDNAs and related information.
(TIF)

Table S1 **Public sequence datasets used in RBFLDB.**
(PDF)

Table S2 **Gene models in each combination of updated structural features.**
(PDF)

Table S3 **Presence and absence profile of cDNAs mapped to** ***Brachypoidum*** **genic regions.**
(PDF)

Table S4 **Presence and absence profile of cDNAs mapped to barley genic regions.**
(PDF)
