# Supplementary figures and images for: Large-Scale Collection and Analysis of Full-Length cDNAs from Brachypodium distachyon and Integration with Pooideae Sequence Resources
Source: PLoS One. 2013 Oct 9;8(10):e75265. doi: 10.1371/journal.pone.0075265 (PMC3793998; doi:10.1371/journal.pone.0075265)

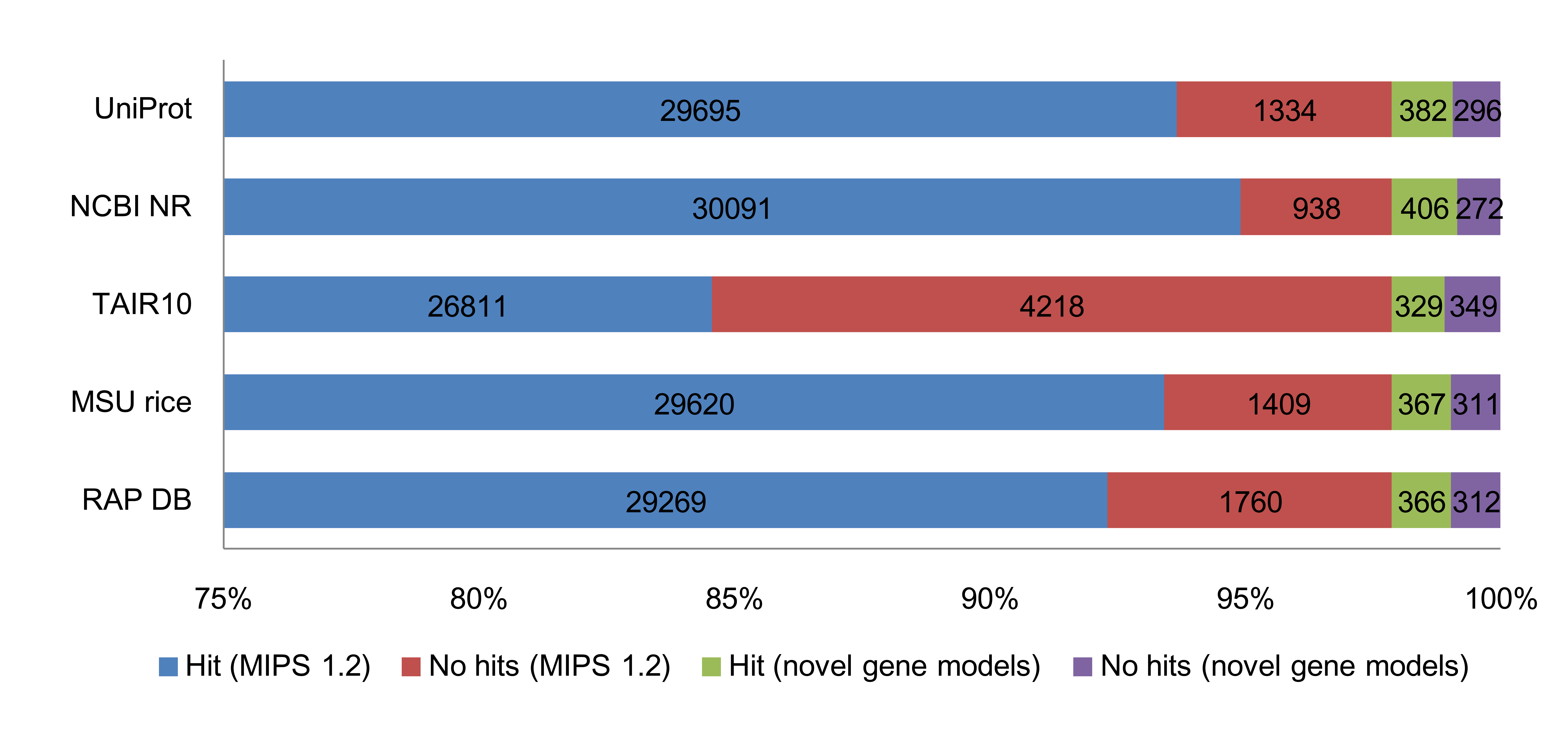

Supplement: Figure S2 — Similarity search results of Brachypodium gene models, including newly identified gene models, against various sequence databases. (TIF) [file pone.0075265.s002.tif]

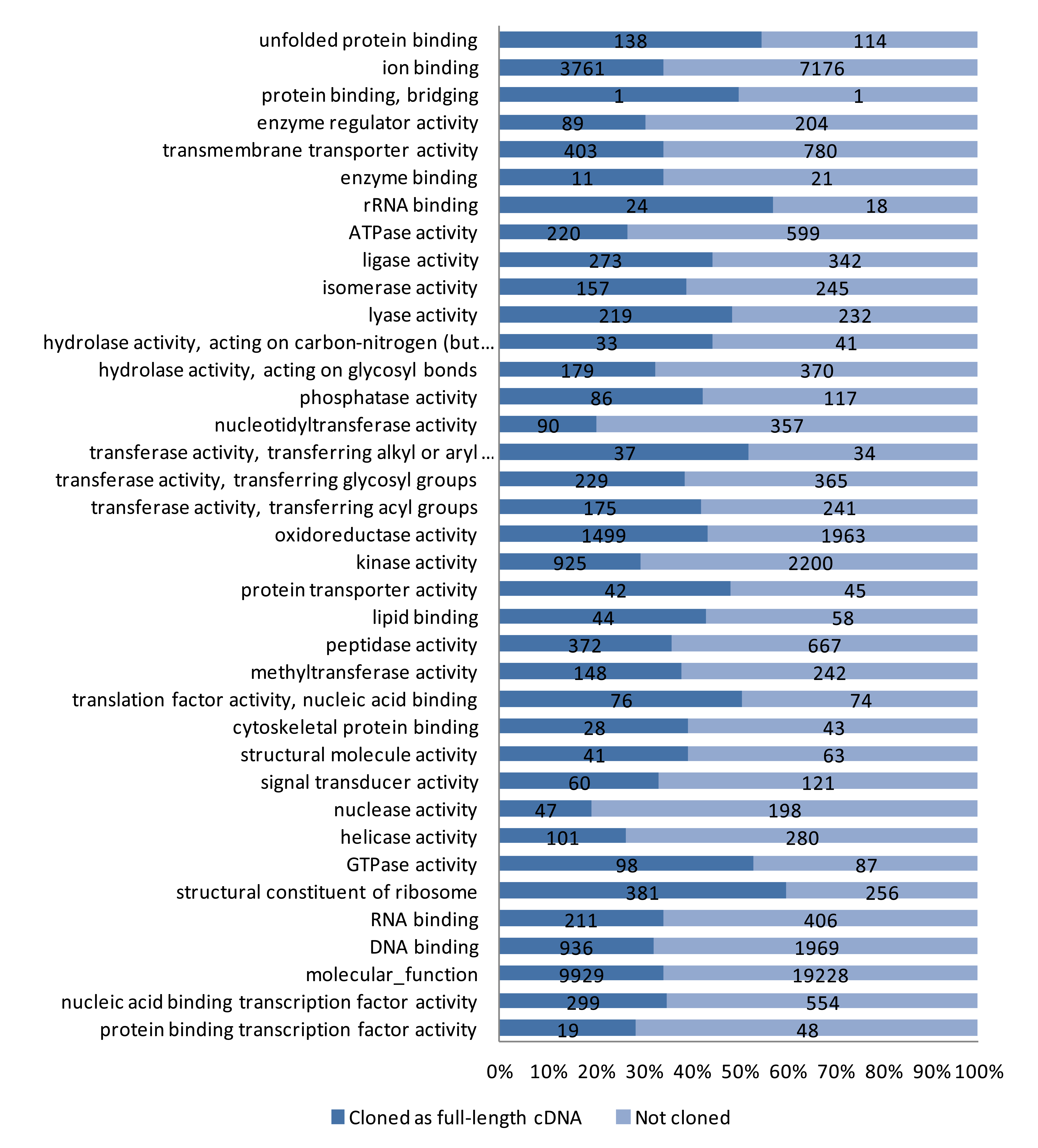

Supplement: Figure S3 — Proportion of Brachypodium genes cloned as full-length cDNAs in a functional classification based on the GO slim category. The represented data is for category in the generic molecular function. (TIF) [file pone.0075265.s003.tif]

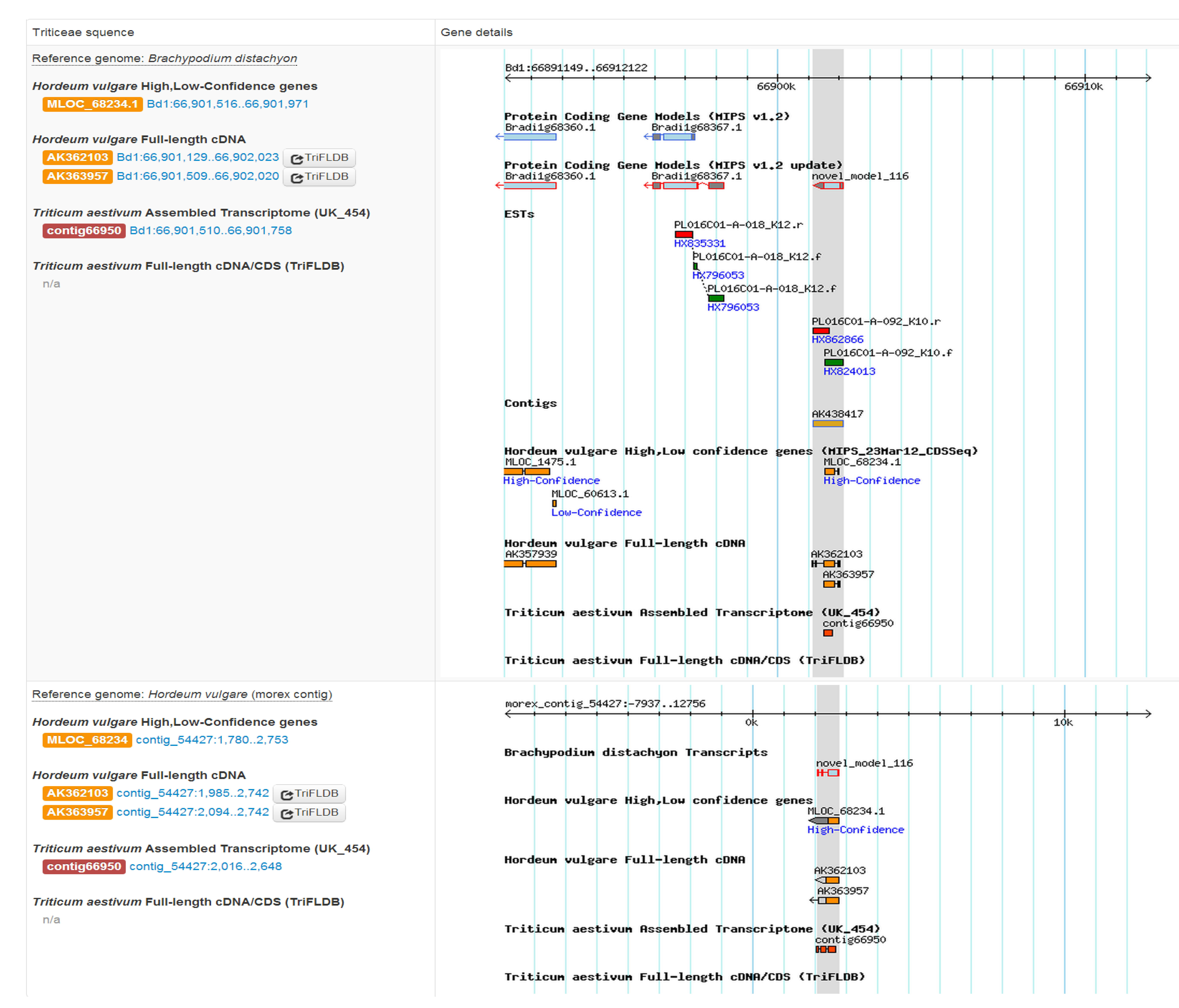

Supplement: Figure S4 — An example of newly identified transcription units based on the RBFL cDNAs, which are supported by homologous cDNAs of barley and wheat. (TIF) [file pone.0075265.s004.tif]
