# Supplementary material for: Large-Scale Collection and Analysis of Full-Length cDNAs from Brachypodium distachyon and Integration with Pooideae Sequence Resources
Source: PLoS One. 2013 Oct 9;8(10):e75265. doi: 10.1371/journal.pone.0075265 (PMC3793998; doi:10.1371/journal.pone.0075265)
Supplement: Table S1 — Public sequence datasets used in RBFLDB. (PDF) [file pone.0075265.s007.pdf]

Supporting Information Table S1. Public sequence datasets used in RBFLDB.

Data type	Dataset	Organism	No. entries	URL	Version	Utility in RBFLDB
Proteome sequence	NCBI nr	all	17,829,843	ftp://ftp.ncbi.nlm.nih.gov/blast/db/	11-Apr-12	Functional prediction
	UniProt (TrEMBL + Swiss-Prot)	all	21,174,559	ftp://ftp.uniprot.org/pub/databases/uniprot/curren t release/knowledgebase/complete/	21-Mar-12	
	TAIR Arabidopsis protein	*Arabidopsis thaliana*	35,386	ftp://ftp.arabidopsis.org/home/tair/Sequences/bla st_datasets/TAIR10_blastsets/	10	
	RAP-DB protein	*Oryza sativa*	42,105	http://rapdb.dna.affrc.go.jp/download/irgsp1.html	2	
	TIGR/MSU Rice protein	*Oryza sativa*	66,338	ftp://ftp.plantbiology.msu.edu/pub/data/Eukaryot ic_Projects/o_sativa/annotation_dbs/pseudomole cules/version 7.0/all.dir/	7	
Genome sequence and annotation	Phytozome 8	*Brachypodium distachyon*	Chromosomes: 83 (including scaffolds)<br>Genes: 26,552<br>mRNAs: 31,029	ftp://ftp.jgi-psf.org/pub/compgen/phytozome/v8.0/Bdistachy on/	8	Annotation update<br>Reference of comparative mapping<br>Genome browser
	MIPS 1.2	*Brachypodium distachyon*	Chromosomes: 83 (including scaffolds)<br>Genes: 26,552<br>mRNAs: 31,029	ftp://brachypodium.org/brachypodium.org/	1.2	
	Barley Morex contig	*Hordeum vulgare*	Contigs: 2,670,738<br>Genes: 75,258<br>mRNAs: 131,447	ftp://ftpmips.helmholtz-muenchen.de/plants/barley/public_data/	3	Reference of comparative mapping<br>Genome browser
	Barley (Ensemble Plants)	*Hordeum vulgare*	Contigs: 289,664<br>Genes: 24,212<br>mRNAs: 62,241	ftp://ftp.ensemblgenomes.org/pub/plants/release-16/gtf/hordeum_vulgare/	030312v2	
cDNAs sequence	Tae FLcDNA (TriFLDB)	*Triticum aestivum*	19,175	http://trifldb.psc.riken.jp/v3/download.pl	3	Queries of comparative mapping
	Hvu FLcDNA (Genbank + TriFLDB)	*Hordeum vulgare*	23,614	http://barleyflc.dna.affrc.go.jp/hvdb/	Acc. AK353559 – AK377172	
			7,341	http://trifldb.psc.riken.jp/v3/download.pl	3	
	Triticum aestivum cDNAs (UK_454)	*Triticum aestivum*	97,481	ftp://ftpmips.helmholtz-muenchen.de/plants/wheat/UK_454/	11-Dec-12	
	Barley Morex cDNAs	*Hordeum vulgare*	131,447	ftp://ftpmips.helmholtz-muenchen.de/plants/barley/public data/	21-Nov-12	
