# Supplementary material for: Large-Scale Collection and Analysis of Full-Length cDNAs from Brachypodium distachyon and Integration with Pooideae Sequence Resources
Source: PLoS One. 2013 Oct 9;8(10):e75265. doi: 10.1371/journal.pone.0075265 (PMC3793998; doi:10.1371/journal.pone.0075265)
Supplement: Table S2 — Gene models in each combination of updated structural features. (PDF) [file pone.0075265.s008.pdf]

Supporting Information TableS2. Gene models in each combination of updated structural features.

| 5'UTR | CDS | 3'UTR | Phytozome 8.0 | MIPS1.2 |
|---|---|---|---|---|
| Extention | Modification | Extention | 32 | 29 |
| Extention | Modification | Addition | 2 | 4 |
| Extention | Modification | Others | 31 | 34 |
| Extention | Modification | | 45 | 46 |
| Addition | Modification | Extention | 98 | 97 |
| Addition | Modification | Addition | 106 | 106 |
| Addition | Modification | Others | 30 | 28 |
| Addition | Modification | | 245 | 244 |
| Others | Modification | Extention | 16 | 15 |
| Others | Modification | Addition | 2 | 2 |
| Others | Modification | Others | 23 | 19 |
| Others | Modification | | 31 | 35 |
| | Modification | Extention | 18 | 18 |
| | Modification | Addition | 13 | 13 |
| | Modification | Others | 7 | 8 |
| | Modification | | 20 | 19 |
| Extention | | Extention | 324 | 321 |
| Extention | | Addition | 38 | 37 |
| Extention | | Others | 23 | 22 |
| Extention | | | 1,543 | 1,528 |
| Addition | | Extention | 431 | 419 |
| Addition | | Addition | 569 | 569 |
| Addition | | Others | 14 | 12 |
| Addition | | | 1,729 | 1,720 |
| Others | | Extention | 8 | 13 |
| Others | | Others | 37 | 23 |
| Others | | | 22 | 17 |
| | | Extention | 544 | 539 |
| | | Addition | 235 | 236 |
| | | Others | 5 | 7 |
| | Modified | | 6,241 | 6,180 |
| | Not modified | | 24,768 | 24,829 |
| | Total | | 31,009 | 31,009 |
