# Supplementary material for: Large-Scale Collection and Analysis of Full-Length cDNAs from Brachypodium distachyon and Integration with Pooideae Sequence Resources
Source: PLoS One. 2013 Oct 9;8(10):e75265. doi: 10.1371/journal.pone.0075265 (PMC3793998; doi:10.1371/journal.pone.0075265)
Supplement: Table S3 — Presence and absence profile of cDNAs mapped to Brachypoidum genic regions. (PDF) [file pone.0075265.s009.pdf]

Supporting Information Table S3.

Hvu FLcDNA	Hvu Morex cDNA	Tae FLcDNA	wheat cDNAs (UK 454)	No. Brachypodium genes
+	+	+	+	8,177
+	+	+	-	804
+	+	-	+	3,872
+	+	-	-	1,674
+	-	+	+	148
+	-	+	-	74
+	-	-	+	130
+	-	-	-	199
-	+	+	+	1,040
-	+	+	-	347
-	+	-	+	1,709
-	+	-	-	1,869
-	-	+	+	219
-	-	+	-	199
-	-	-	+	594
with at least one				**21,055**
-	-	-	-	5,839
Total				26,894

Hvu FLcDNA: barley full-length cDNAs
Hvu Morex cDNA: gene models annotated in the barley Morex genome
Tae FLcDNA: wheat full-length cDNAs
wheat cDNA(UK454): wheat gene models of a shotgun genome assembly
