# Supplementary material for: Large-Scale Collection and Analysis of Full-Length cDNAs from Brachypodium distachyon and Integration with Pooideae Sequence Resources
Source: PLoS One. 2013 Oct 9;8(10):e75265. doi: 10.1371/journal.pone.0075265 (PMC3793998; doi:10.1371/journal.pone.0075265)
Supplement: Table S4 — Presence and absence profile of cDNAs mapped to barley genic regions. (PDF) [file pone.0075265.s010.pdf]

Supporting Information Table S4.
Presence and absence profile of cDNAs mapped to barley genic regions.

Bdi transcript	Hvu FLcDNA	Tae FLcDNA	wheat cDNA (UK 454)	No. barley genes
+	+	+	+	5,891
+	+	+	-	699
+	+	-	+	3,221
+	+	-	-	1,668
+	-	+	+	2,392
+	-	+	-	489
+	-	-	+	2,542
+	-	-	-	2,632
-	+	+	+	960
-	+	+	-	324
-	+	-	+	1,581
-	+	-	-	4,557
-	-	+	+	901
-	-	+	-	527
-	-	-	+	4,023
with at least one cDNAs				**32,407**
-	-	-	-	42,851
Total				75,258

Bdi Transcript: Brachypodium transcripts
Hvu FLcDNA: barley full-length cDNAs
Tae FLcDNA: wheat full-length cDNAs
wheat cDNA (UK454): wheat gene models of a shotgun genome assembly
